# Causal effects of gut microbiota on the risk of osteomyelitis: a Mendelian randomization study

**DOI:** 10.3389/fmicb.2024.1342172

**Published:** 2024-05-28

**Authors:** Ran Xu, Si Li, Ying Zhang, Yue Pu, Guangcheng Luo, Xinjun Wang

**Affiliations:** ^1^Department of Urology, Zhongshan Hospital Xiamen University, School of Medicine, Xiamen University, Xiamen, China; ^2^Department of Pediatric Surgery, Women and Children’s Hospital, School of Medicine, Xiamen University, Xiamen, China; ^3^Department of Urology, Zhongshan Hospital Xiamen University, The School of Clinical Medicine, Fujian Medical University, Xiamen, China

**Keywords:** gut microbiota, osteomyelitis, Mendelian randomization, causal relationship, MiBioGen

## Abstract

**Background:**

Osteomyelitis is characterized by an inflammatory process initiated by microorganisms, leading to infection and subsequent degradation of bone tissue. Several studies have indicated a potential link between gut microbiota and the occurrence of osteomyelitis. Utilizing the benefits of Mendelian randomization, which mitigates issues of confounding and reverse causation, we employed this approach to ascertain the presence of a causal connection between gut microbiota and osteomyelitis. Additionally, we aimed to pinpoint gut microbiota that could potentially exert substantial influence.

**Methods:**

We performed a rigorous screening of single nucleotide polymorphisms in GWAS summary statistics for gut microbiota and osteomyelitis. The 2,542 instrumental variables obtained after screening were subjected to MR analyses, including inverse variance weighting, weighted median, weighted mode, MR-Egger, and Mendelian randomization pleiotropy residual sum and outlier test. We then validated the reliability of the results by performing sensitivity analyses on the MR of 196 well-defined gut microbiota.

**Result:**

We established a causal relationship between gut microbiota and osteomyelitis through MR analysis. Additionally, we identified a taxon of significant importance and six taxons with nominal significance. Specifically, the family Bacteroidales S24.7 group exhibited an association with a diminished risk of osteomyelitis development. Conversely, the class Bacilli, class Bacteroidia, order Bacteroidales, order Lactobacillales, family Streptococcaceae, and genus Coprococcus3 displayed an increased risk of developing osteomyelitis. The MR outcomes for these seven taxa remained stable throughout a series of sensitivity analyses.

**Conclusion:**

This study demonstrated a causal relationship between gut microbiota and osteomyelitis by Mendelian randomization. We hope that this study will provide a new direction for the treatment of osteomyelitis, which has a paucity of therapeutic options.

## 1 Introduction

Osteomyelitis represents an inflammatory process in which microorganisms incite infection leading to subsequent bone degradation (Lew and Waldvogel, 2004). This condition manifests in three predominant forms: osteomyelitis resulting from microorganism migration from an adjacent source (such as trauma-induced cases), osteomyelitis arising secondarily due to vascular insufficiency or neuropathy (typified by diabetic foot ulcers), and acute hematogenous osteomyelitis. Notably, *Staphylococcus aureus* stands as the primary causative microorganism behind osteomyelitis occurrences (Peltola et al., 2010). The mortality rate associated with osteomyelitis can escalate to as much as 8%, thus imposing a significant burden on global public health systems (Kremers et al., 2015; Urish and Cassat, 2020). The central therapeutic approaches encompass prolonged antibiotic regimens and surgical debridement. Despite the implementation of these interventions, patients retain a heightened propensity for developing persistent infections or sustaining concurrent health conditions. Consequently, there exists a justified impetus to explore novel treatment modalities for osteomyelitis.

The gut microbiota comprises a diverse array of microorganisms residing within the human gastrointestinal tract. Research has illuminated that the gut microbiota plays a multifaceted role, encompassing human immune regulation, metabolic processes, and various other activities. Furthermore, it exhibits a close correlation with metabolic disorders, autoimmune conditions, and sundry diseases (Lawlor et al., 2008; Maynard et al., 2012; Lee et al., 2020). Notably, recent observational investigations have underscored a significant interplay between alterations in gut microbiota composition and metabolism, and the progression of osteomyelitis (Bui et al., 2022). While these findings imply a robust connection between gut microbiota and osteomyelitis, it’s crucial to acknowledge that observational studies are susceptible to confounding factors and the issue of reverse causation. Hence, to procure more dependable outcomes, we employed Mendelian randomization to deduce the relationship between gut microbiota and osteomyelitis.

The gut microbiota could potentially influence osteomyelitis through various mechanisms. Firstly, Interleukin 1β (IL-1β), one of the two forms of IL-1, is initially produced as an inactive protein and subsequently secreted through protease-mediated cleavage. This process activates the IL-1 receptor (IL-1R), initiating inflammatory signaling (Kanneganti, 2010; Lukens et al., 2012). Studies have demonstrated that gut microbiota play a role in the development of osteomyelitis by enhancing the expression of IL-1β (Lukens et al., 2014; Phillips et al., 2016). Secondly, the microbe-rich gut environment results in a significant upregulation of polyamines (Ramos-Molina et al., 2019). These polyamines contribute to the regulation of T cell progression, the promotion of macrophage polarization, and the reduction of pro-inflammatory cytokine production, such as TNF-α (Proietti et al., 2020). Furthermore, polyamines hinder the differentiation of osteoclasts, consequently mitigating bone loss (Yamamoto et al., 2012). Research has provided supportive evidence for the potential involvement of polyamines in osteomyelitis (Bui et al., 2022). While these findings imply a robust connection between gut microbiota and osteomyelitis, it’s crucial to acknowledge that observational studies are susceptible to confounding factors and the issue of reverse causation. Hence, to procure more dependable outcomes, we employed Mendelian randomization to deduce the relationship between gut microbiota and osteomyelitis.

The concept of Mendelian randomization was initially introduced by Katan (1986). Their argument rested on the principle of random allele assignment during gamete formation, suggesting that genotypes could serve as instrumental variables for studying intermediate phenotypes. This approach facilitates the inference of causal connections with disease states while mitigating the influence of confounders and reverse causality on effect estimates. Consequently, a prominent advantage of Mendelian randomization is its resilience against confounding factors including behavioral, social, psychological influences, and reverse causality.

Employing summary statistics derived from a extensive genome-wide association study (GWAS) dataset, we applied Mendelian randomization to ascertain the presence of a causal link between gut microbiota and osteomyelitis, while also discerning potentially impactful gut microbiota.

## 2 Materials and methods

### 2.1 Study design

**Figure 1:**
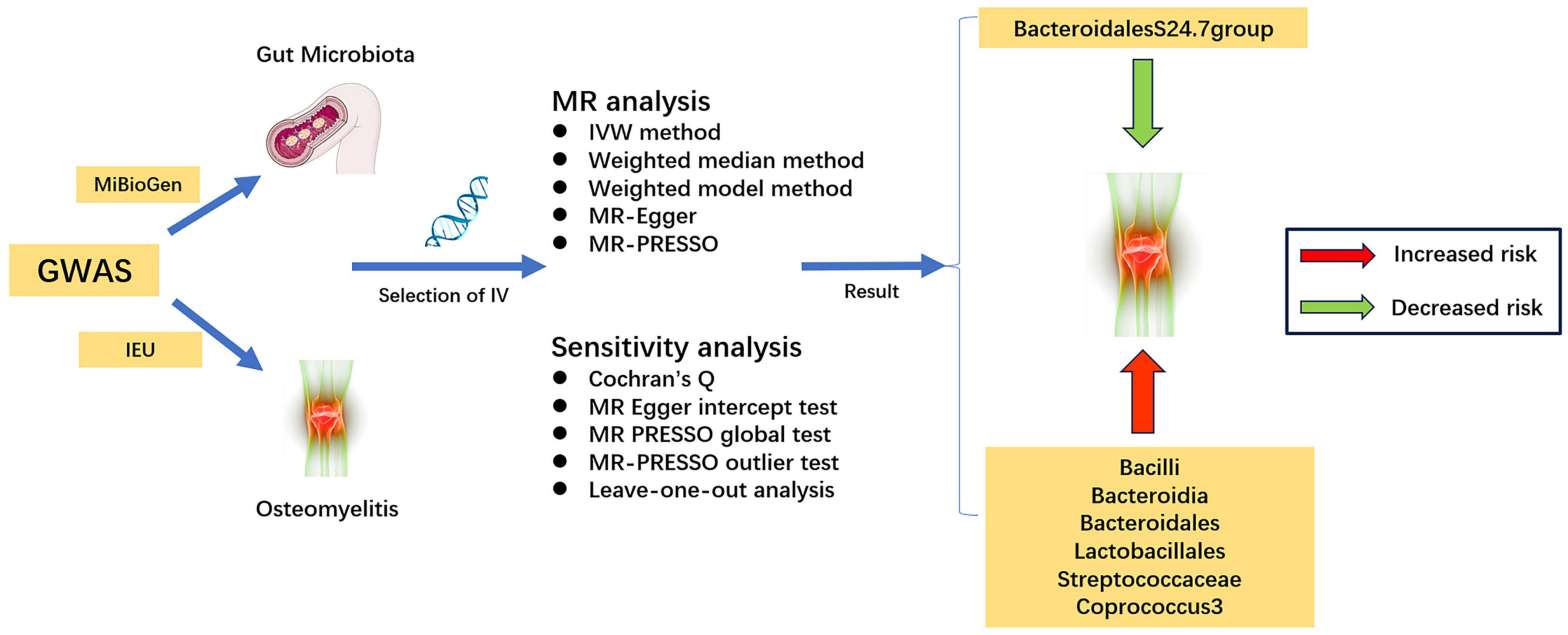
The study’s overall flowchart.

The flowchart of the study is illustrated in Figure 1. Three core assumptions must be met in Mendelian randomization studies: (1) instrumental variables should exhibit correlation with exposure factors, (2) instrumental variables must remain uncorrelated with confounders, and (3) instrumental variables should exclusively influence the outcome variable via exposure factors (Bowden and Holmes, 2019).

### 2.2 Data sources

The gut microbiota GWAS data were sourced from the MiBioGen consortium’s repository^1^. Our study encompassed a diverse cohort of 18,340 participants hailing from 11 distinct countries. To ensure the highest standards of precision, we elected to omit 15 taxa due to ambiguous definitions. Consequently, our analysis focused solely on 196 well-defined gut microbiota taxa (9 at the phylum level, 16 at the order level, 20 at the family level, 32 at the order level, and 119 at the genus level), as detailed in Supplementary Table S1 (Kurilshikov et al., 2021). For our investigation into osteomyelitis, GWAS summary statistics were retrieved from the IEU GWAS database^2^. This specific analysis comprised 4,836 cases of European origin and 481,648 matched controls, all originating from European populations.

### 2.3 Selection of instrumental variables

To acquire robust instrumental variables tightly linked with exposure factors, an essential step involves the scrutiny of single nucleotide polymorphisms (SNPs) within the gut microbiota GWAS dataset. The quantity of instrumental variables incorporated in the MR analysis significantly impacts the subsequent MR outcomes. Augmenting the count of instrumental variables not only bolsters the statistical robustness of the MR findings but also introduces greater heterogeneity into these outcomes (Sekula et al., 2016).

In this study, we established a screening threshold of *p* < 1 × 10^−5^ for instrumental variable selection. This particular threshold was chosen for its capacity to harmonize the trade-off between statistical power and the heterogeneity of MR outcomes. Moreover, it’s notable that this threshold has been adopted in numerous MR investigations centered around the gut microbiota (Liu et al., 2023; Zeng et al., 2023).

To ensure the independence of each instrumental variable, we applied criteria that excluded those with an *r*^2^ value below 0.001 and a genetic distance exceeding 10,000 kb, effectively mitigating potential linkage disequilibrium (Zeng et al., 2023). Furthermore, we eliminated palindromic SNPs and SNPs not represented in the dataset results. In addition, SNPs exhibiting a strength below *F* < 10 were omitted from instrumental variable selection due to the susceptibility of weak instrumental variables to bias (Burgess and Thompson, 2011). The *F* statistic was employed as a metric for gaging the strength of the instrumental variables, calculated by the formula *F* = *β*^2^ exposure/SE^2^ exposure (Emdin et al., 2017; Zhang et al., 2022).

Finally, we took steps to exclude SNPs associated with risk factors for the outcome, as identified by PhenoScanner, from the pool of instrumental variables.

### 2.4 Mendelian randomization analysis and sensitivity analysis

The Mendelian randomization (MR) analysis in this study employed a variety of methods, including the Inverse-Variance-Weighted (IVW) method, Weighted Median method, Weighted Model, MR-Egger, and the Mendelian Randomization Pleiotropy Residual Sum and Outlier (MR-PRESSO) test. The IVW method utilizes Wald estimators and deltas to compute ratio estimates for individual Single Nucleotide Polymorphisms (SNPs), which are then aggregated to derive primary causal effect estimates (Burgess et al., 2013). Among the considered methods, IVW exhibited superior precision in effect estimation, prompting its selection as the primary analytical approach. The other techniques were employed for supplementary validation of IVW findings (Bowden et al., 2016; Yavorska and Burgess, 2017). Additionally, statistical power calculations for causal effect estimates were performed using the MR online power calculation tool^3^ (Burgess, 2014).

To mitigate potential issues of invalid instrument bias and pleiotropy associated with the Inverse Variance Weighted method, this study undertook sensitivity analyses to assess the validity and robustness of IVW results. Firstly, we quantified and assessed potential heterogeneity in MR analysis outcomes using Cochran’s *Q* test. Secondly, we evaluated the presence of horizontal pleiotropy through the MR Egger intercept test and the MR-PRESSO global test, with statistical significance set at *p* < 0.05 (Burgess and Thompson, 2017). The MR-PRESSO outlier test was employed with 10,000 distributions to identify and remove outliers, thereby addressing horizontal pleiotropy (Verbanck et al., 2018). Concurrently, the impact of individual abnormal SNPs on Mendelian randomization results was appraised using leave-one-out analysis.

### 2.5 Statistical analysis

To ensure more robust conclusions, we employed the Bonferroni method to adjust the *p*-values (VanderWeele and Mathur, 2019). Given that each taxonomic level (phylum, class, order, family, and genus) encompasses multiple gut microbial taxa, the adjusted threshold for each level is set at 0.05/N. Here, *N* stands for the count of taxa within the respective level. Accordingly, the adjusted thresholds for phylum, class, order, family, and genus levels are: 5.56 × 10^−3^ (0.05/9), 3.13 × 10^−3^ (0.05/16), 2.50 × 10^−3^ (0.05/20), 1.56 × 10^−3^ (0.05/32) and 4.20 × 10^−4^ (0.05/119). Results from Mendelian randomization (MR) showing *p*-values below the Bonferroni-adjusted threshold were deemed significant. MR results with *p*-values <0.05 were regarded as nominally significant (Liu et al., 2023; Luo et al., 2023). A *p*-value below 0.05 was deemed statistically significant for the sensitivity analysis in this investigation. We expressed the link between gut microbiota and bipolar disorder in terms of an odds ratio (OR) accompanied by its corresponding 95% confidence interval (CI).

All of the above analyses were primarily performed using the Two-Sample-MR package (version 0.5.7) with R software (version 4.2.3).

## 3 Results

### 3.1 Details of IVs

After a rigorous screening process as described in the previous narrative, a total of 2,542 SNPs were finally included as instrumental variables in this study (Supplementary Table S2).

### 3.2 MR analysis

**Figure 2:**
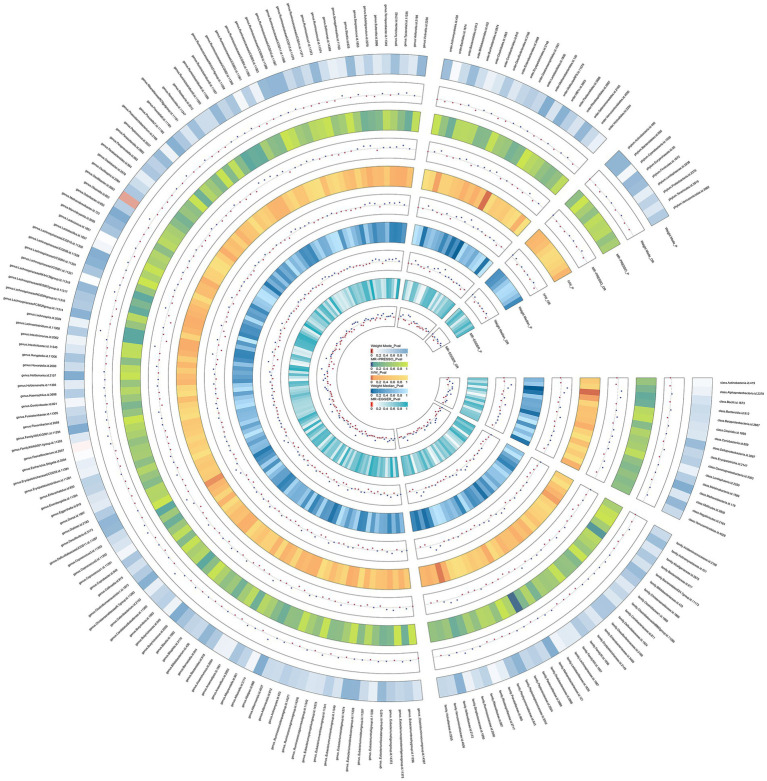
Preliminary MR estimates for the associations between gut microbiota and the risk of osteomyelitis. From the inner to outer circles, they represent the estimates of: MR-Egger, weighted median, inverse-variance weighted methods, MR-PRESSO and weighted mode, re-spectively. And the shades of color reflect the magnitude of the *p*-value.

The results of the preliminary analysis of the relationship between the gut microbiota and osteomyelitis are shown in Figure 2 and Supplementary Table S3.

**Figure 3:**
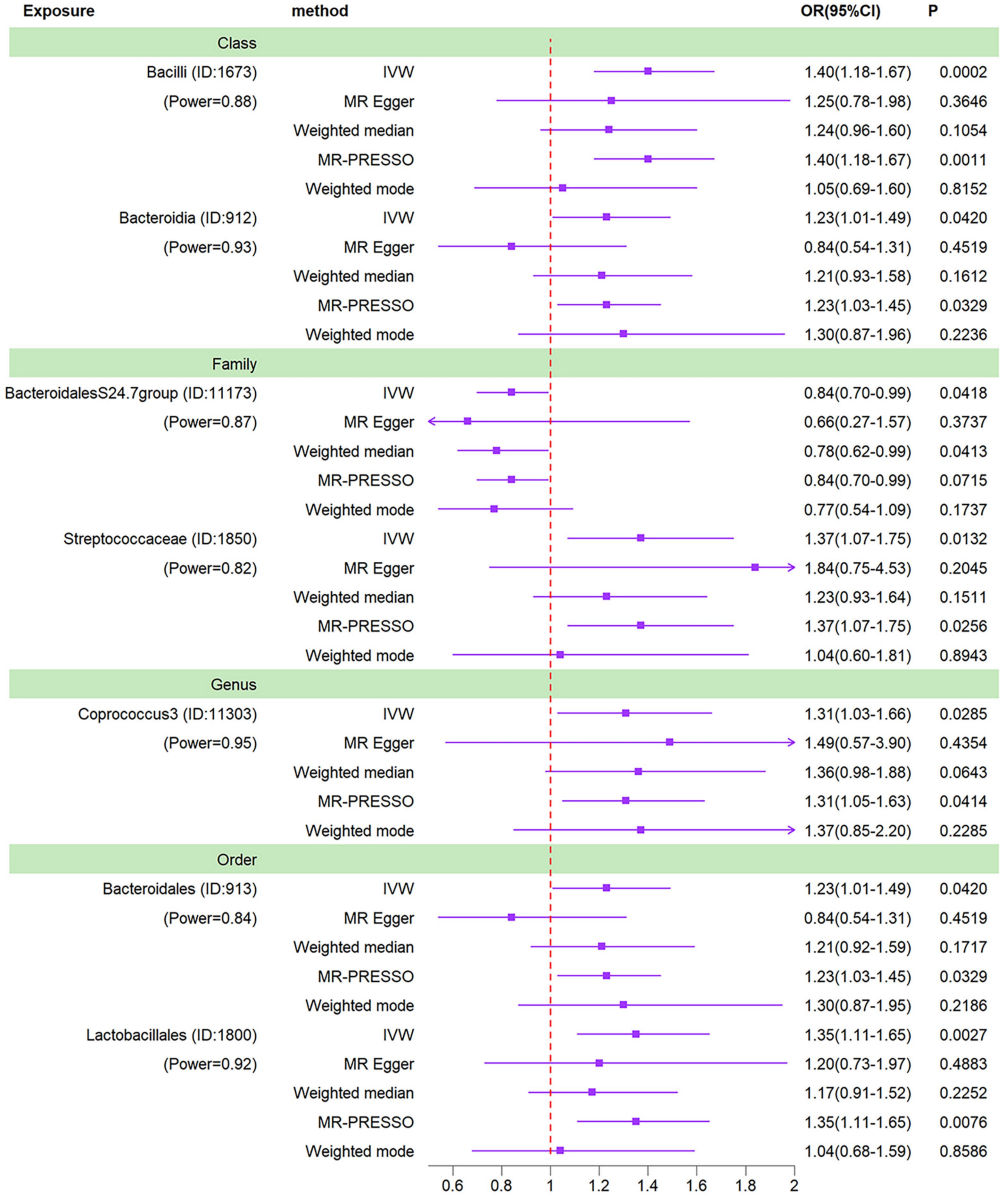
Forest plots of MR results for seven gut microbiotas on osteomyelitis. Method, statis-tical analysis methods; OR, odds ratio; 95% CI, 95% confidence interval; *p*, significance *p*-value; Power, the statistical power of causal effect estimates.

We conducted a Mendelian randomization (MR) analysis on a cohort of 196 well-defined gut microbiota using the Inverse Variance Weighting (IVW) method as the primary analytical approach. Our analysis revealed a total of seven taxa exhibiting *p*-values below 0.05 in the MR results. Notably, one among these taxa, specifically the class Bacilli, displayed a *p*-value for MR outcomes that remained significant even after applying the Bonferroni correction threshold. This finding led us to identify one gut microbiota with significant impact and six others with nominally significant effects (Figure 3). In relation to osteomyelitis influence, we observed that the family BacteroidalesS24.7group (OR = 0.84, 95% CI = 0.70–0.99, *p* = 0.0418) was associated with a reduced risk of osteomyelitis development. Conversely, the class Bacteroidia (OR = 1.23, 95% CI = 1.01–1.49, *p* = 0.0420), order Bacteroidales (OR = 1.23, 95% CI = 1.01–1.49, *p* = 0.0420), genus Coprococcus3 (OR = 1.31, 95% CI = 1.03–1.66, *p* = 0.0285), order Lactobacillales (OR = 1.35, 95% CI = 1.11–1.65, *p* = 0.0027), family Streptococcaceae (OR = 1.37, 95% CI = 1.07–1.75, *p* = 0.0132), and class Bacilli (OR = 1.40, 95% CI = 1.18–1.67, *p* = 0.0002) exhibited associations with an increased risk of osteomyelitis development.

Of these 7 gut microbiota, there was a lack of parallelism between MR-Egger and IVW results for 2 taxa (class Bacteroidia and order Bacteroidales). Typically, under such circumstances, it would necessitate a reassessment of the instrumental variables using stricter thresholds (Chen et al., 2021). Nonetheless, these two taxa are already characterized by a limited number of instrumental variables, making it challenging to ensure the statistical robustness of the MR outcomes should the count of instrumental variables be diminished further. A threshold of 1 × 10–5 is the standard choice for the majority of Mendelian randomization investigations concerning the gut microbiota (Yu et al., 2023; Zeng et al., 2023). Furthermore, the IVW analysis exhibited greater precision in estimating causal effects compared to the MR-Egger analysis. Lastly, a similar scenario was noted in the study conducted by Luo et al., where the decision was made to retain taxa for which the MR-Egger outcomes did not align with the IVW results (Luo et al., 2023). Consequently, given the absence of heterogeneity and horizontal pleiotropy, it was deemed acceptable to retain the IVW results pertaining to these two taxa.

### 3.3 Sensitivity analysis

**Figure 4:**
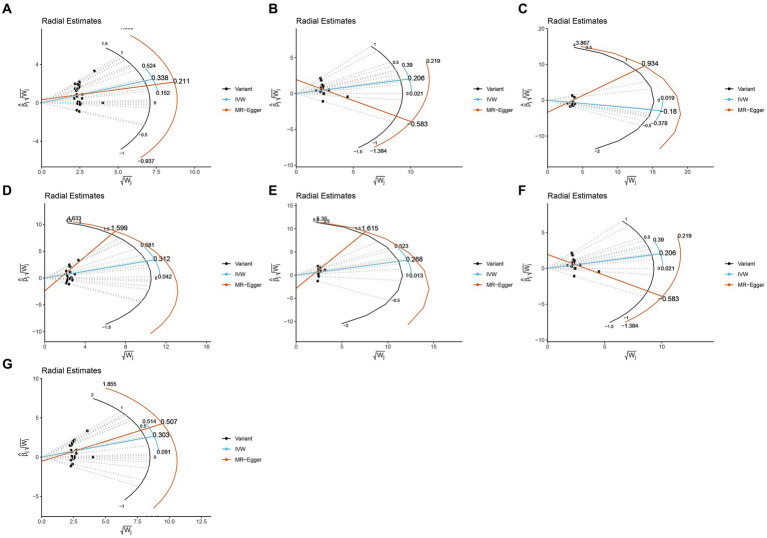
Radial plots from one significant and six nominally significant gut microbiota taxa as-sociated with osteomyelitis. **(A)** Class Bacilli; **(B)** Class Bacteroidia; **(C)** Family Bacteroi-dalesS24.7group; **(D)** Family Streptococcaceae; **(E)** Genus Coprococcus3; **(F)** Order Bacteroidales; **(G)** Order Lactobacillales.

The study assessed the potential impact of invalid instrumental bias or pleiotropy on MR results by conducting sensitivity analyses to ensure the validity and robustness of the findings. Among the seven gut microbial taxa that exhibited significant or nominal significance, none displayed heterogeneity according to Cochran’s *Q* test (Table 1). This observation was visually supported by the funnel plot (Supplementary Figure S1). Additionally, both the MR-Egger intercept test and the MR-PRESSO global test yielded *p*-values exceeding 0.05, indicating the absence of horizontal pleiotropy-induced effects on the MR results. The examination of radial plots (Figure 4) and leave-one-out analyses (Supplementary Figure S2) confirmed the absence of outliers or abnormal SNPs, thus establishing the reliability of the MR results for the seven gut microbial taxa. Supplementary Figures S3, S4 provide visual representations of the remaining analyses.

**Table 1:** Sensitivity analysis for one significant and six nominally significant gut microbiota taxa associated with osteomyelitis.

Exposure	Outcome	Cochran’s Q	Cochran’s Q pval	Egger_intercept	Egger_intercept pval	MR-PRESSO global test pval
Bacilli	Osteomyelitis	25.00	0.25	0.01	0.59	0.26
Bacteroidia	Osteomyelitis	11.24	0.74	0.03	0.08	0.71
BacteroidalesS24.7group	Osteomyelitis	8.94	0.44	0.02	0.60	0.45
Streptococcaceae	Osteomyelitis	24.60	0.06	−0.02	0.51	0.07
Coprococcus3	Osteomyelitis	7.63	0.57	−0.01	0.79	0.63
Bacteroidales	Osteomyelitis	11.24	0.74	0.03	0.08	0.71
Lactobacillales	Osteomyelitis	24.58	0.14	0.01	0.61	0.16

MR-PRESSO, Mendelian randomization pleiotropy residual sum and outlier.

## 4 Discussion

We provided a comprehensive assessment of the causal relationship between 196 gut microbiota and osteomyelitis using MR analysis. The results showed that there is indeed a causal relationship between gut microbiota and osteomyelitis. In addition, we identified one taxon of significance and six taxons of nominal significance. The family BacteroidalesS24.7group was associated with a reduced risk of developing osteomyelitis, the class Bacilli, the class Bacteroidia, the order Bacteroidales, the order Lactobacillales, the family Streptococcaceae and the genus Coprococcus3 were associated with an increased risk of osteomyelitis. The MR results for these seven taxa remained stable in a series of sensitivity analyses. Our findings are reliable and provide new ideas for the treatment of osteomyelitis.

In a conducted study, Tina et al. discovered that oligofructose impacts the severity of osteomyelitis through the modification of gut microbiota composition and metabolism (Bui et al., 2022). Specifically, they observed a correlation between Bifidobacteria presence and a decreased risk of osteomyelitis development. In contrast, a separate research by Phillips et al. revealed that Prevotella exacerbates osteomyelitis severity, while Lactobacillus mitigates it (Phillips et al., 2016). The observations made by Tina and the findings of Phillips et al. diverge from the influential gut microbiota we have identified. This incongruity between clinically observed outcomes and genetically predicted results could arise from intricate interactions within the gut microbiota. To address this discrepancy, thorough validation necessitates further prospective randomized controlled trials.

We are the first MR study to demonstrate a causal association between gut microbiota and osteomyelitis from a genetic prediction perspective. In addition, we identified seven significant gut microbiota that provide potential research targets for the prevention and treatment of osteomyelitis. Our study also has limitations. The GWAS data on osteomyelitis used in this study did not provide detailed individual information, and we were unable to perform stratified subgroup analyses of osteomyelitis subtypes. In addition, most of the participants in the GWAS data used in this study were of European origin, so it remains unknown whether the findings can be generalized to other non-European populations.

## 5 Conclusion

This study demonstrated a causal relationship between gut microbiota and osteomyelitis by Mendelian randomization. We hope that this study will provide a new direction for the treatment of osteomyelitis, which has a paucity of therapeutic options.

## Data availability statement

The original contributions presented in the study are included in the article/Supplementary material, further inquiries can be directed to the corresponding author.

## Ethics statement

No additional ethical approval is required as this is a re-analysis of data that is already publicly available.

## Author contributions

RX: Data curation, Formal analysis, Methodology, Software, Visualization, Writing – original draft, Writing – review & editing. SL: Data curation, Software, Visualization, Writing – review & editing. YZ: Data curation, Software, Visualization, Writing – review & editing. YP: Data curation, Methodology, Visualization, Writing – review & editing. GL: Conceptualization, Data curation, Formal analysis, Methodology, Software, Writing – original draft, Writing – review & editing. XW: Conceptualization, Data curation, Formal analysis, Investigation, Methodology, Software, Visualization, Writing – original draft, Writing – review & editing.

## Footnotes

^1^ https://mibiogen.gcc.rug.nl

^2^ https://gwas.mrcieu.ac.uk/

^3^ https://shiny.cnsgenomics.com/mRnd/
