# Supplementary material for: Causal effects of gut microbiota on the risk of osteomyelitis: a Mendelian randomization study
Source: Front Microbiol. 2024 May 28;15:1342172. doi: 10.3389/fmicb.2024.1342172 (PMC11166080; doi:10.3389/fmicb.2024.1342172)
Supplement: Supplementary file 1 [file Table_1.DOCX]

Supplementary Material

Causal effects of gut microbiota on the risk of osteomyelitis: a Mendelian randomization study

***Ran Xu^1^, Si Li^1^, Ying Zhang^2^, Yue Pu^1^, Guangcheng Luo^1, 2^ , Xinjun Wang^1, 2*^***

*Corresponding author: Xinjun Wang，Department of Urology，Zhongshan Hospital Xiamen University. No. 201-209, Hubin South Road, Siming District, Xiamen, Fujian, 361000, China. Tel:86-0592-2292201. Fax:86-0592-2292201. E-mail: wxj@xmu.edu.cn.


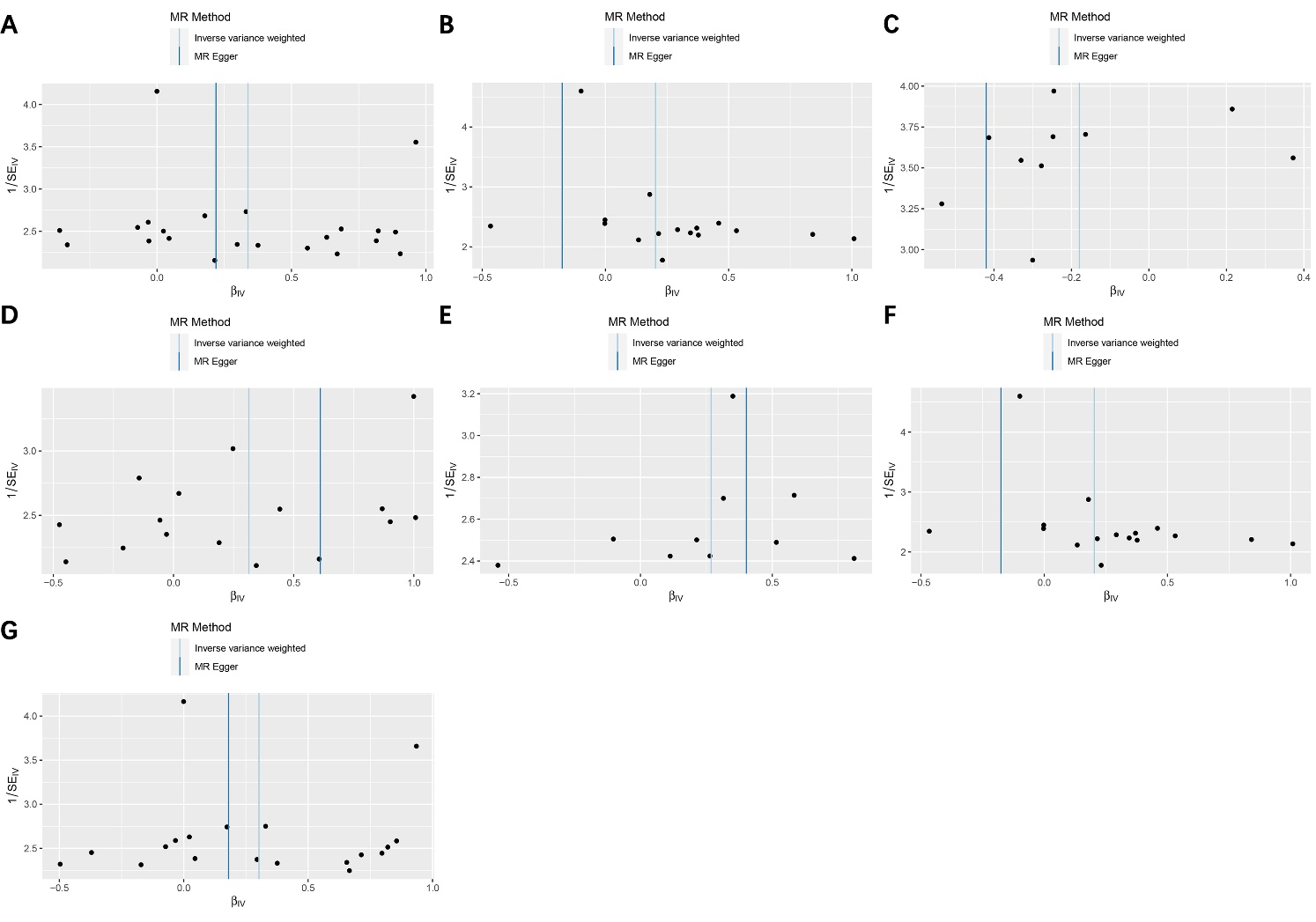


**Figure S1:** Funnel plots from seven gut microbiota taxa on the risk of osteomyelitis. (A) Class Bacilli; (B) Class Bacteroidia; (C) Family BacteroidalesS24.7group; (D) Family Streptococcaceae; (E) Genus Coprococcus3; (F) Order Bacteroidales; (G) Order Lactobacillales.


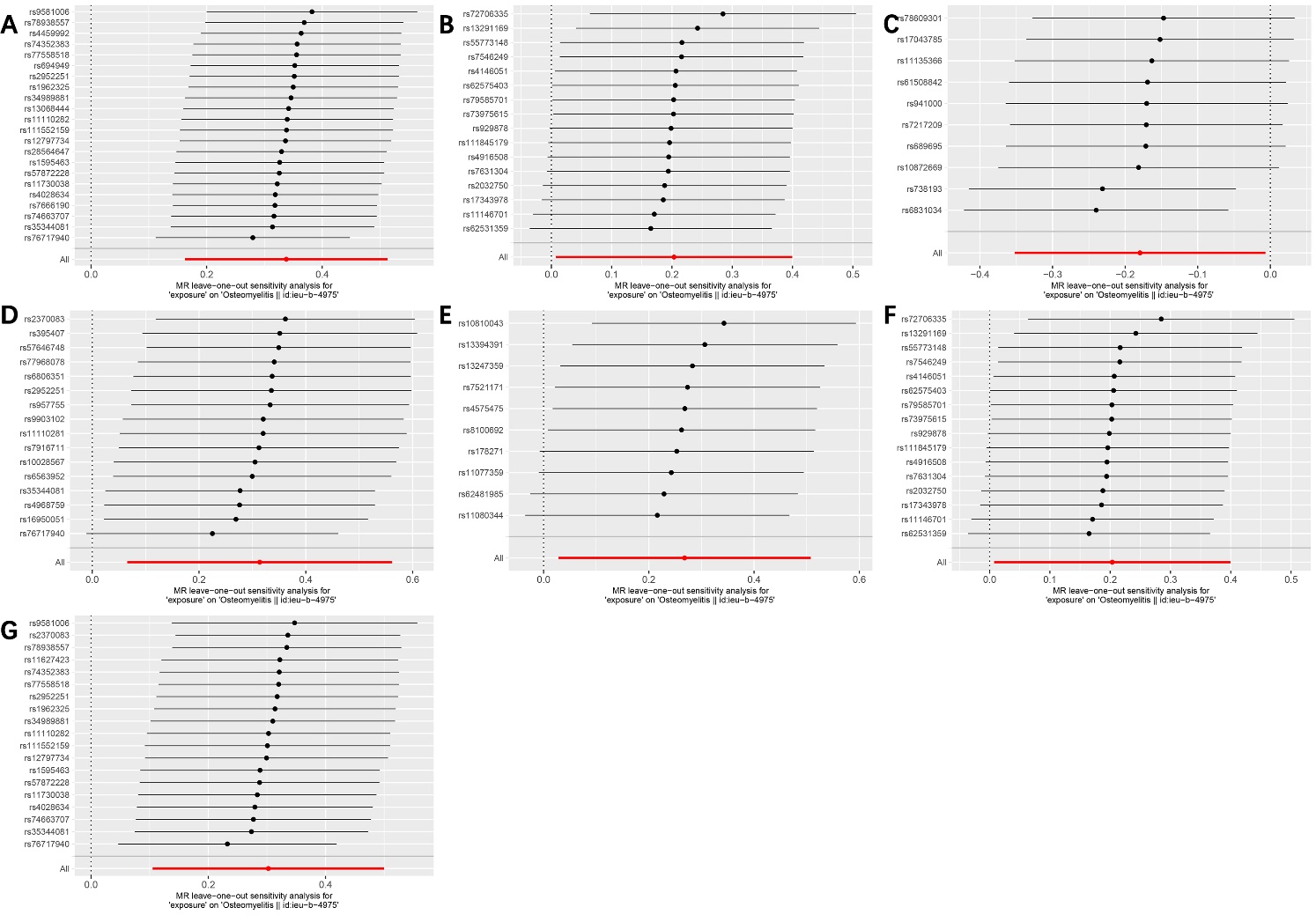


**Figure S2:** Leave-one-out analysis from seven gut microbiota taxa on the risk of osteomyelitis. (A) Class Bacilli; (B) Class Bacteroidia; (C) Family BacteroidalesS24.7group; (D) Family Streptococcaceae; (E) Genus Coprococcus3; (F) Order Bacteroidales; (G) Order Lactobacillales.

**
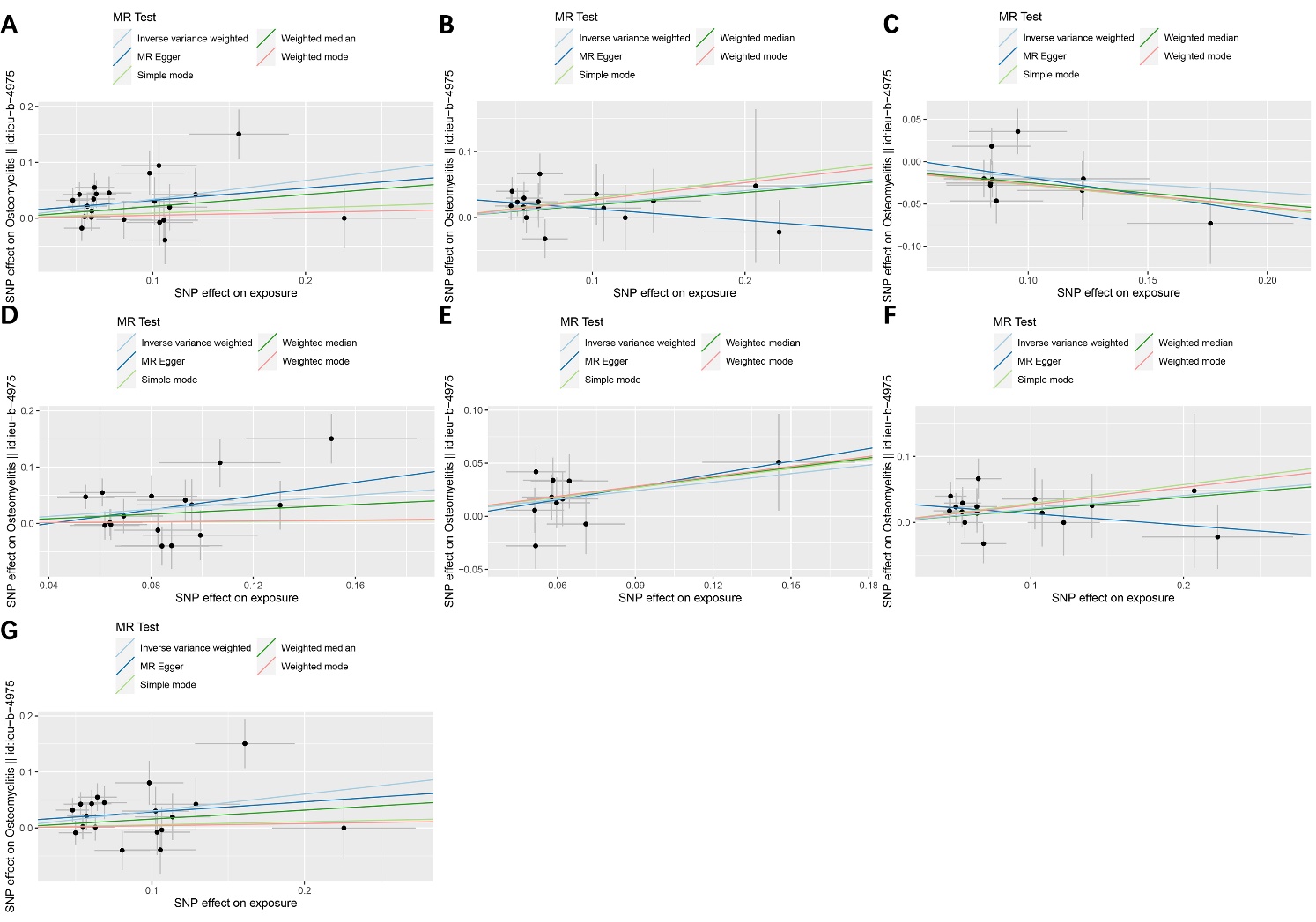
**

**Figure S3:** Scatter plot from seven gut microbiota taxa on the risk of osteomyelitis. (A) Class Bacilli; (B) Class Bacteroidia; (C) Family BacteroidalesS24.7group; (D) Family Streptococcaceae; (E) Genus Coprococcus3; (F) Order Bacteroidales; (G) Order Lactobacillales.


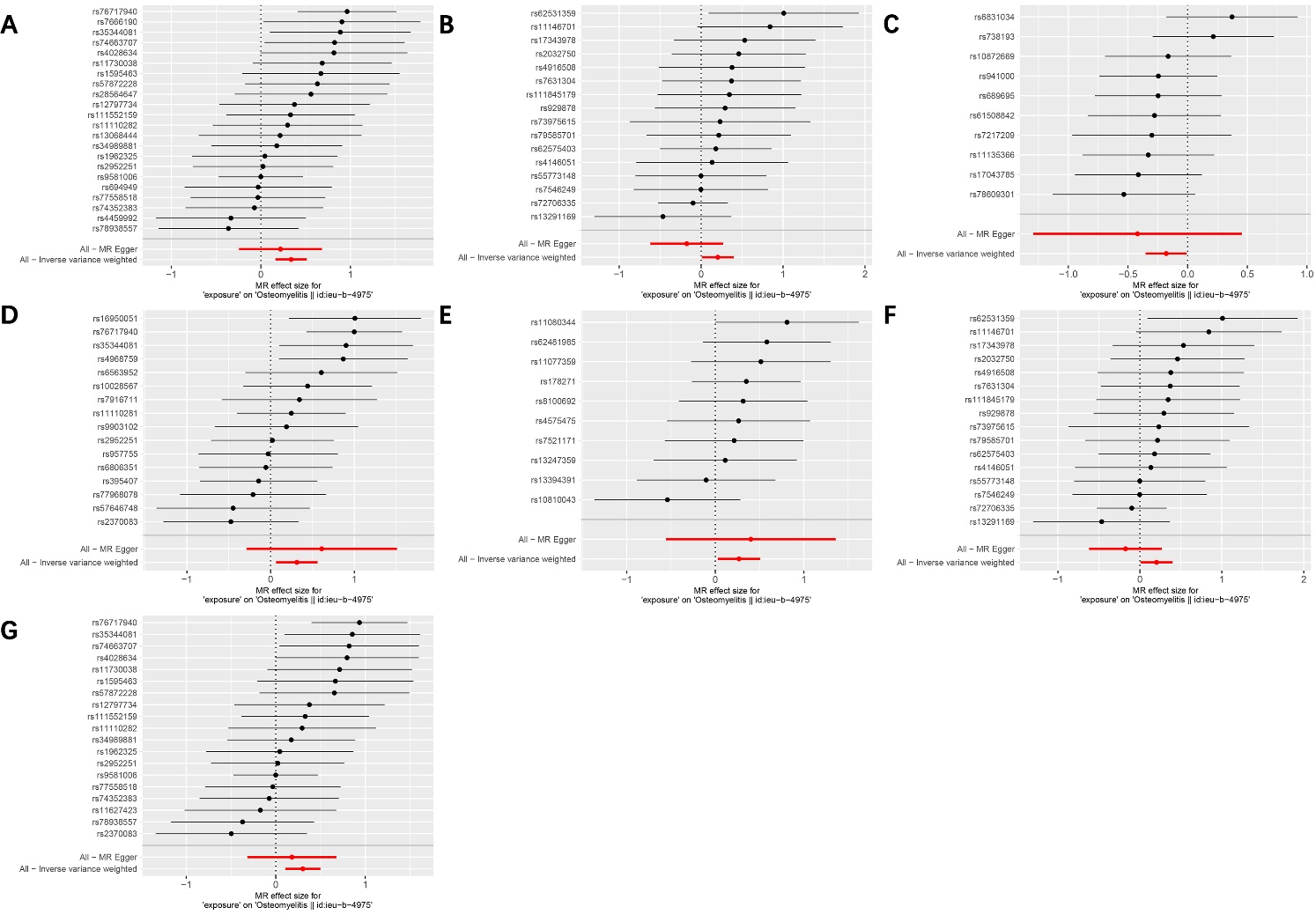


**Figure S4:** Plot from seven gut microbiota taxa on the risk of osteomyelitis. (A) Class Bacilli; (B) Class Bacteroidia; (C) Family BacteroidalesS24.7group; (D) Family Streptococcaceae; (E) Genus Coprococcus3; (F) Order Bacteroidales; (G) Order Lactobacillales.
